# Words and Meters: Neural Evidence for a Connection Between Individual Differences in Statistical Learning and Rhythmic Ability in Infancy

**DOI:** 10.1111/desc.70116

**Published:** 2025-12-31

**Authors:** Iris van der Wulp, Marijn Struiksma (Marijn), Frank Wijnen (Frank)

**Affiliations:** ^1^ Department of Languages, Literature and Communication Institute for Language Sciences, Utrecht University Utrecht the Netherlands

**Keywords:** EEG, individual differences, infants, neural entrainment, rhythmic ability, statistical learning

## Abstract

Music and language are both hierarchically structured: syllables combine into words, and meters are groupings of musical beats. Statistical learning (SL) supports speech segmentation through computation of transitional probabilities between syllables, and individual differences in SL ability were found predictive of further language development. The current study investigated whether rhythmic ability (RA; the ability to perceive the beat and infer the meter in a rhythmic stimulus auditory input) correlated with SL in 6‐ to 9‐month‐old infants. We further explored whether RA of the parents predicted infant RA and/or SL. We used EEG to measure infants’ neural entrainment in two conditions: (1) a speech segmentation SL condition in which transitional probabilities between syllables were the only cue to segment the speech stream into words; (2) a RA condition exposing infants to a syncopated rhythm in a quadruple meter. We correlated neural entrainment to the words in the SL condition with entrainment to the meter in the RA condition. Parents completed behavioral tasks measuring their RA and were asked about their engagement with music. Results revealed a correlation between neural entrainment indexing SL and RA in infants, which was unaffected by infant age. This correlation was specific to neural entrainment to words and meters, suggesting similarity between processing items at hierarchically corresponding levels. We found no evidence that parental RA predicted infant RA or SL. However, frequency of parent–child joint musical engagement appeared to have a positive effect on infant RA.

## 1 Introduction

### 1.1 Individual Differences in Statistical Learning Predicting Language Acquisition

*Speech segmentation* (segmenting continuous speech into words) is an important and challenging first step in language acquisition, as word boundaries are not consistently marked in continuous speech. *Statistical learning* (SL) is hypothesized to support speech segmentation (Saffran, Aslin, et al. 1996) and entails tracking *transitional probabilities* (TPs) between (successive) syllables. TPs refer to the probability that an element *X* is followed by *Y*, given the overall frequency of *X* (Saffran et al. 1996). TPs in most Western languages are higher for syllable transitions within words than between words (Saffran 2003, but see also Gervain and Guevara Erra 2012; Saksida et al. 2017 for language‐specificity accounts), and therefore useful as cues for detecting likely word boundaries. Experimental research showed that infants can rely on TPs to segment continuous speech in the absence of other (e.g., prosodic) cues (Choi et al. 2020; Fló et al. 2019; Fló et al. 2022; Saffran, Newport, et al. 1996; Singh et al. 2012; i.a.).

**Summary**

- We found evidence for a correlation between measures of neural entrainment indexing statistical learning and rhythmic ability in 6‐ to 9‐month‐old infants.
- Neural entrainment was unaffected by infant age.
- We did not find evidence that parental rhythmic ability was a predictor of child rhythmic ability or statistical learning.
- Indices of parent–child joint musical engagement provided results in a positive direction, which pave the way for further studies on this topic.

SL performance in infancy is associated with outcome measures of subsequent language development (Isbilen and Christiansen 2022; Kidd et al. 2018; Newman et al. 2016; Siegelman 2020; Singh et al. 2012). In addition, several studies point to a SL deficit in individuals whose language development is disordered (e.g., Evans et al. 2009; Kerkhoff et al. 2013; Lammertink et al. 2017; Vandermosten et al. 2019). This evidence suggests that individual differences in SL are associated with variations in language acquisition trajectories.

However, the neurocognitive bases of such individual differences in SL are still unclear. Can other cognitive mechanisms be identified that interact with SL, and are they present and identifiable early in life? If so, we gain insight into the interindividual variability of language development trajectories and do so at an age when early intervention is possible. The present study investigated individual differences in SL in infants by evaluating a new hypothesis, that musical rhythmic ability (RA) is one cognitive mechanism that (co‐)determines individual variations in SL ability. In order to test this hypothesis, we need to avail of a measure of SL that is both sensitive to individual variability and suitable for the infant population.

### 1.2 Methodological Advancements in Assessing SL in Infants

Experiments investigating SL typically consist of a familiarization and a test phase. In the familiarization phase, participants hear a continuous stream of spoken syllables constructed by concatenating a limited number of multisyllabic pseudowords in a pseudorandom order, as illustrated in (1a) (Saffran, Aslin, et al. 1996). TPs for syllables within each pseudoword is 1.0, whereas the TP for syllables at word boundaries is lower (0.33 in example (1a)).

1. a. **Familiarization Phase**

… ***tupiro**golabubidakupadotigolabubidaku**tupiro**padoti*…

b. **Test Phase**: head‐turn preference paradigm

*tupiro* (word). versus *tigola* (foli).

Experiments investigating SL in infants mostly employ looking times as measured with a *head‐turn preference procedure* (HPP) as an outcome measure (Saffran, Aslin, et al. 1996; Singh et al. 2012; i.a.). In the HPP (example 1b), differences in looking times between familiar “words” (patterns with high TPs presented during familiarization) and foils (syllable sequences presented in the familiarization that include a low TP signaling a word boundary) have been taken to indicate that infants succeeded in segmenting words on the basis of TP information. The HPP is conducted *after* the familiarization phase, thus providing at best an indirect measure of the SL process (e.g., Isbilen and Christiansen 2022).

In contrast to the HPP, *entrainment* of neural oscillations (henceforth “neural entrainment”), as measured with *electroencephalography* (EEG) provides a direct window on the “online” detection of TP‐patterns and is not convoluted by the subsequent formation of memory representations (Batterink and Paller 2017; Choi et al. 2020; Fló et al. 2022). By keeping the presentation rate of spoken syllables constant, a corresponding frequency is present in the auditory signal (e.g., 3.3 Hz for syllables of 300 ms). Upon processing these stimuli, the same frequency becomes dominant in the EEG. If participants track TPs and thus detect word boundaries, a multi‐syllabic word frequency (i.e., one step up in the hierarchy) that is not present in the auditory stimuli, but needs to be inferred based on the TPs, will arise in the EEG signal as well (1.1 Hz for tri‐syllabic words of 900 ms). More neural entrainment to the word frequency was found to indicate more successful speech segmentation, correlating with behavioral measures of (implicit) memory of the word forms in both adults (Batterink and Paller 2017; van der Wulp et al. 2025), and infants (Choi et al. 2020). As both identification of the TPs and the formation of word‐form memory representations are subject to inter‐individual variation, neural entrainment is a measure of SL that is more sensitive to individual differences in the process of tracking TPs than the HPP (Batterink and Paller 2017).

### 1.3 RA Supporting SL

We propose that interindividual differences in SL are related to differences in RA; defined as an individual's ability to perceive the beat and recognize the meter in a rhythmic auditory input, with corresponding neural or behavioral rhythmic synchronization with these external (musical) rhythms. Our hypothesis is built on several research findings establishing (1) correlations between RA and language development, and (2) evidence for overlap in neurocognitive mechanisms underlying both RA and SL.

#### 1.3.1 Correlations Between RA and Language Development

Similar to language, musical rhythm is hierarchically organized, where beats are at the level of sensory perception, from which a higher‐level *meter* can be induced: a top‐down inferred grouping of beats (Lenc et al. 2021).^1^ A person's propensity for neural entrainment to beat and meter frequencies can be used to index their RA (Di Liberto et al. 2020; Fiveash et al. 2021). There is behavioral and neural evidence that infants as young as 5 months are able to infer the meter from various rhythmic stimuli (Cirelli et al. 2016; Hannon and Johnson 2005; Lenc et al. 2022). RA has been shown to (longitudinally) predict linguistic development (Fiveash et al. 2021; Ladányi et al. 2020). Moreover, parents’ musical experience (having had ≥5 years of musical training) as well as infant music classes predicted better infant RA indicated by more neural entrainment to beat and meter frequencies (Cirelli et al. 2016).

#### 1.3.2 Neurocognitive Overlap Between Linguistic SL and RA

In addition, there is evidence for shared neural mechanisms processing musical rhythm and language. In an experiment by Assaneo et al. (2019), adult participants who spontaneously synchronized their speech to the isochronous rhythm of a spoken syllable sequence (“high synchronizers”) performed better in a behavioral SL speech segmentation task than “low synchronizers”, who did not show this auditory‐motor synchronization. High synchronizers also showed more neural entrainment while listening to speech than low synchronizers. Furthermore, the degree of left‐dominant hemispheric asymmetry—measured by the fractional anisotropy^2^ of the arcuate fasciculus—was larger in high synchronizers compared to low synchronizers. The arcuate fasciculus is the white matter tract that connects the dorsal auditory processing stream (Hickok and Poeppel 2007), which runs left‐lateralized from the auditory to the motor cortex, continuing to the inferior frontal gyrus (including Broca's area, specifically Brodmann area 44). The dorsal stream is involved in processing of both language and music (Cannon and Patel 2021; Lenc et al. 2022; Patel and Iversen 2014; Vaquero et al. 2018). Although high synchronizers had more years of musical experience than low synchronizers, the authors found that years of musical training did not (fully) explain the group membership results (Assaneo et al. 2019, 5). This points to the possibility of RA as an inherited ability independent of musical training. As musical ability was found to be heritable (e.g., Gingras et al. 2015; Niarchou et al. 2022), we hypothesized that the dorsal stream is part of the neurological substrate of not only language, but also innate musical ability, which would show individual variation already from birth.

### 1.4 Current Study

The objective of the present study was to address the following research questions:

1. Do infants with better RA also show better SL abilities when both are measured using neural entrainment?
2. Does parental RA predict infant RA abilities?
3. Does parental RA predict infant SL abilities?

**FIGURE 1:**
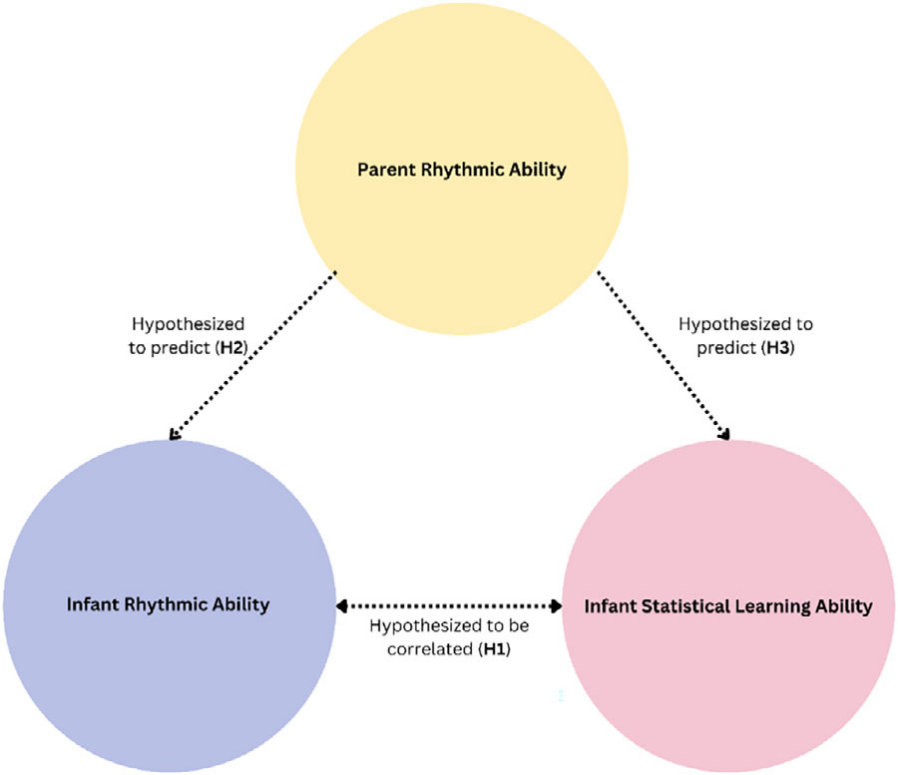
Visualization of the predictions that were made in this study.

We investigated if SL‐based speech segmentation ability in infants between 6 and 9 months of age correlated with their RA. We predicted that the degree of neural entrainment to the meter in the RA condition correlates positively with the degree of neural entrainment to the words in the SL condition (**H1**, Figure 1), pointing to a neurocognitive overlap between RA and SL that is already effective in early childhood.

Additionally, we explored whether parental RA predicts infant RA and SL abilities. If RA is heritable, we expected that an infant's rhythm processing is predicted their parents’ rhythmic/musical ability (**H2**). Finally, considering the hypothesized connection between SL and RA, we predicted that parental RA and musicality would also predict infant SL (**H3**, Figure 1).

## 2 Methodology

### 2.1 Participants

A total of 44 infants (28 males) and their parents participated in this study. The infants’ ages ranged between 6;20 (months; days) and 9;05 (*M *= 7;27, *SD *= 0;20). The 6‐to‐9 months age range was based on earlier studies attesting SL and RA in infancy, using neural entrainment at different ages (newborn: Fló et al. 2022; 6 months: Choi et al. 2020; Lenc et al. 2022; 7 months: Cirelli et al. 2016; (behavioral) SL at 8 months: Saffran, Aslin, et al. 1996).

Choosing an age range increased the number of families that were able to participate. We investigated possible contributions of age to all reported effects.

The families were recruited via the participant database of the Utrecht University Institute for Language Sciences Babylab, located in the city of Utrecht, the Netherlands. All infants were growing up with Dutch as their native language. We excluded families from participation if the child was being raised multilingually, and if a parent was diagnosed with Developmental Dyslexia, Developmental Language Disorder, or another language‐related impairment. We also excluded families from participating if the infant had ever had an ear infection, or any other hearing problems, neurological disorders, or was born preterm at 32 weeks or less. Parents in our sample were highly educated (University: 68 (80%); Applied University: 16 (18.8%): High School: 1 (1.2%)).

Informed consent was provided by the parents for the participation of the child as well as for themselves. Upon completion of the tasks at home (Section 2.3.2), the parents individually received a gift card with a value of €5. After participation in the lab, the infant received a children's book, and parents were able to get reimbursement of travel costs (up to €5), if applicable.

#### 2.1.1 Excluded and Missing Data

One infant only completed the RA condition. One infant removed the EEG cap before the minimum exposure was reached in the first condition presented, leaving no usable EEG data. Furthermore, we excluded data from two infants because excessive artifacts prevented the Artifact Blocking algorithm (Mourad et al. 2007; Section 2.4.1.) from estimating the smoothing matrix. Our logbook and visual inspection of the data indicated that both these infants had pulled the cables of the EEG apparatus repeatedly during the experiment. Thus, 40 infants were included in the SL condition, and 41 in the RA condition.

Eighty‐five out of 88 parents completed all tasks (42 families completed all tasks with both parents). One family consisted of two mothers, of which only the genetically related mother completed the tasks. In the case of one other family, one parent completed the questionnaire, while the other completed the PROMS. Neither of them completed the CA‐BAT (Section 2.3.2).

### 2.2 Stimuli

#### 2.2.1 SL Condition

**FIGURE 2:**
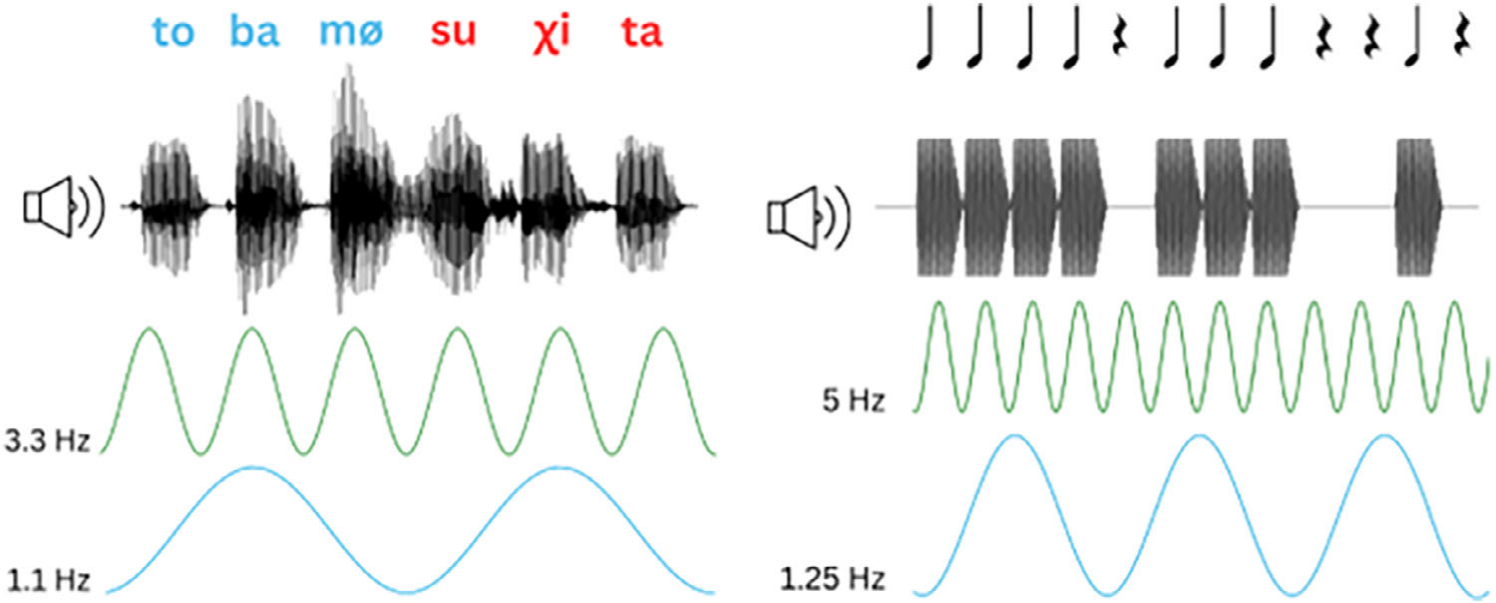
Stimuli in the SL condition (left), and RA condition (right), with the respective phonetic and musical scores, audio waveform, syllable/beat frequencies (green waves), and word/meter frequencies (blue waves).

The stimuli in the SL condition were identical to two blocks of the stimuli used in an experiment reported in van der Wulp et al. (2025). They consisted of CV‐syllables combined into four tri‐syllabic pseudowords adhering to Dutch phonotactic constraints: /suχita, tobamø, sytøbo, χøbyti/. The TPs between syllables were 1.0 within words and 0.33 between words. The pseudowords were concatenated into two 4.5‐min speech streams, in which each pseudoword was repeated 75 times (300 words; 900 syllables). This yielded a stream consisting of 600 words (1800 syllables) with a total duration of 9 min. The word order was pseudorandomized, such that the same word did not consecutively repeat. All syllables had a 300 ms duration (consonant = 100 ms; vowel = 200 ms) yielding a syllable frequency of 3.3 Hz, and word length of 900 ms and corresponding word frequency of 1.1 Hz (Figure 2).

Speech was generated with the MBROLA synthesizer, using the male Dutch voice *nl2* at a monotone F0 of 100 Hz (Dutoit et al. 1996). We used GoldWave (GoldWave Inc. 2022) to add cue points^3^ to the audio for each syllable onset, which served as EEG markers. A linear, 1.5 s fade‐in and fade‐out was applied to the beginning and the end of the speech stream, to avoid presenting a confounding segmentation cue. For more information on how these stimuli were created, see van der Wulp et al. (2022).

#### 2.2.2 RA Condition

We took the stimulus for the RA condition from Lenc et al. 2018; Lenc et al. 2022).^4^ This consists of a three‐measure *syncopated* rhythm (i.e., a weakly periodic rhythm, created by omitting beats in metrically strong positions). Each measure comprised four counts (either expressed as tones or silent periods), hence yielding a quadruple (4/4) meter. In the first measure, all four tones were played. In the second measure, the first tone was omitted while the other three were played. In the third measure, only the third tone was played and the first, second and final counts of the measure were filled with silent periods (Figure 2). The beats and silent periods lasted 200 ms each, with four beats/silent periods per measure (quadruple meter; one measure lasting 200 × 4 = 800 ms). This corresponds to a beat frequency of 5 Hz and a meter frequency of 1.25 Hz. The rhythmic pattern lasted 2.4 s in total and was repeatedly presented in two blocks of 4 min, yielding 100 repetitions of the rhythmic pattern per block. In total, this corresponds to 8 min of stimuli in the RA condition, comprising 200 repetitions of the rhythmic pattern (600 m, consisting of 2400 beats and silent period events).

Lenc et al. (2022) showed that 5‐ to 6‐month‐old infants neurally entrained to the meter‐related frequencies of this rhythm. They also showed that the meter in this syncopated rhythm is not readily perceivable from the acoustic properties of the stimulus, by comparing their infant EEG data to a cochlear model that only responded to the acoustic properties of the stimulus without top‐down cognition. They found that the response to the meter frequency was significantly larger in the infant EEG than can be expected from the acoustic properties of the rhythmic pattern alone. The authors concluded based on this difference between the cochlear model and the EEG data that the representation of the meter does not simply arise from properties of the stimulus, but is internally represented in the infant brain. We used their low‐tone stimuli, as they had observed that this enhanced rhythm perception in both periodic and syncopated conditions compared to delivering these stimuli with a high tone.

The RA stimuli were played by a 130 Hz tone with 10 ms rise time and 50 ms fall linear ramps (Lenc et al. 2022). We used GoldWave (GoldWave Inc. 2022) to add cue points to the audio for each beat/silent period onset (every 200 ms), which served as EEG markers, similar to the cue points in the SL condition. We also included a linear fade‐in and fade‐out of 1.5 s each in this condition, to make the RA condition more comparable to the SL condition.

### 2.3 Procedure

#### 2.3.1 Infant EEG Session

**FIGURE 3:**
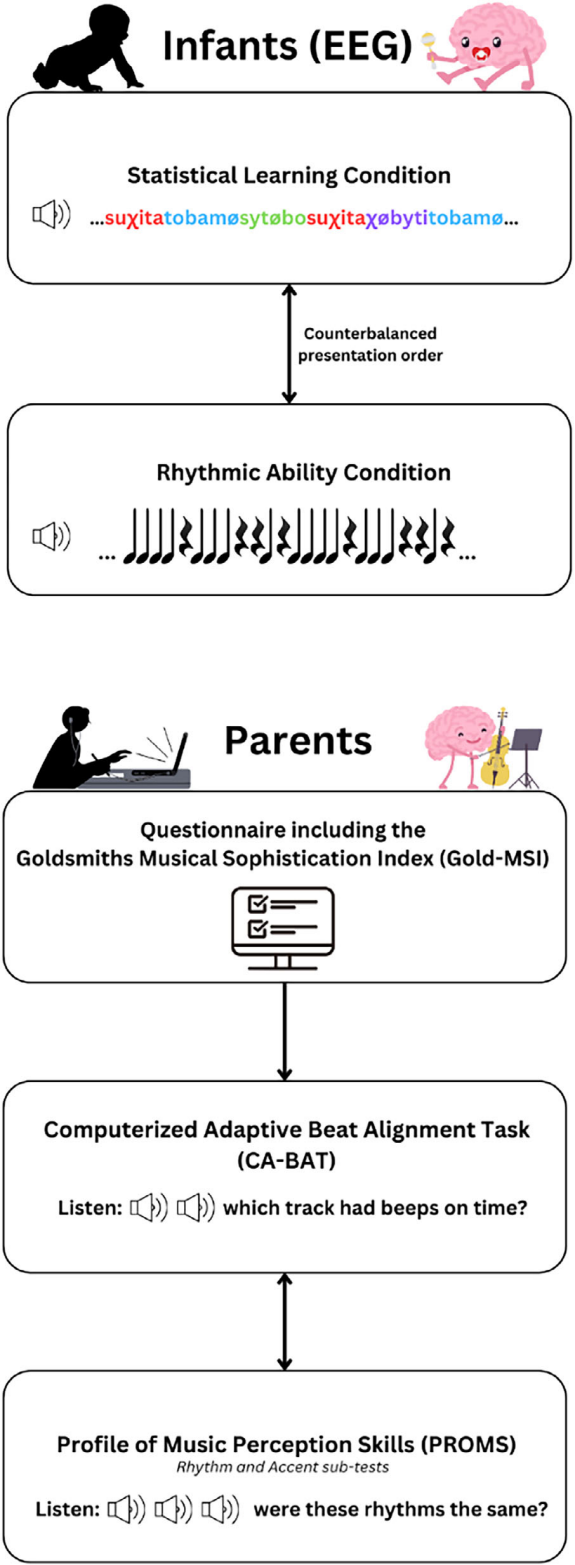
Visual representation of the experimental procedure.

In the lab, EEG was recorded from the infants while they listened to the SL and RA stimuli subsequently (order SL/RA first was counterbalanced between infants; Figure 3). EEG was recorded at a sampling rate of 512 Hz using 32 Ag/AgCl‐tipped active electrodes attached to an electrode headcap following the 10/20 system. Recordings were made with the Active‐Two system (Biosemi, Amsterdam, The Netherlands) and corresponding ActiView software. Additional electrodes were placed on the left and right mastoids. Scalp signals were recorded online relative to the Common Mode Sense (CMS) electrode. The impedance was accepted below 20 mV, otherwise the channel was classified as bad during data collection.

**FIGURE 4:**
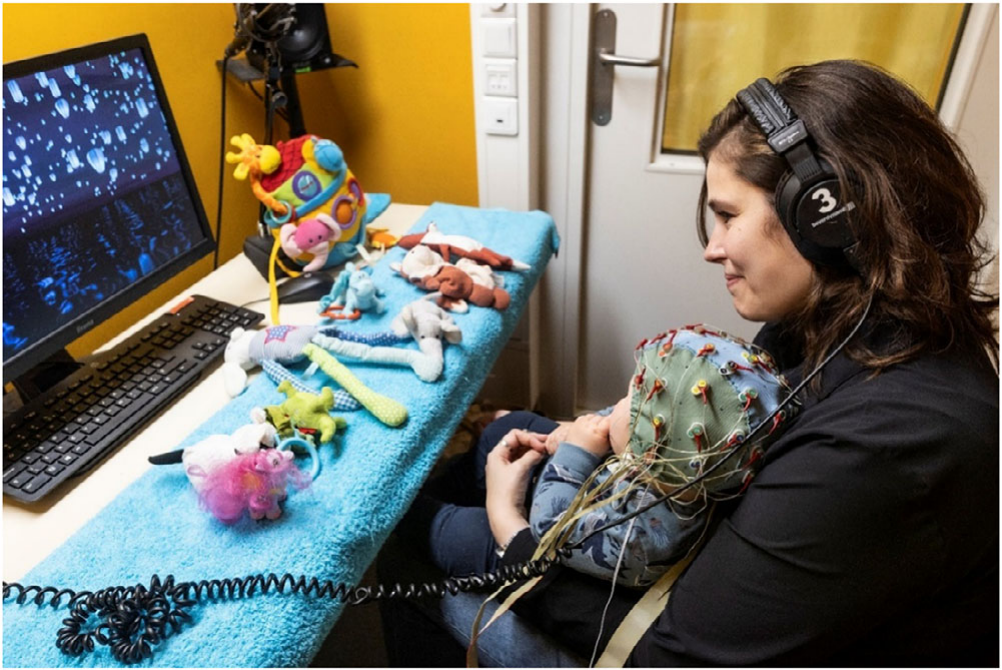
Example of the experimental set‐up during the EEG session in the lab. Picture taken by Dieuwertje Bravenboer.

During listening, the infant was seated on their parent's lap in a sound‐attenuated booth. The audio was played through stereo speakers^5^. The parent was wearing headphones playing masking audio. A video of colorful lanterns that slowly drifted upward^6^ was playing on a screen^7^ in front of the infant. The infants were allowed to play with soft, non‐audible toys during the session. See Figure 4 for a depiction of the experimental set‐up.

In both conditions, we adhered to a minimum and maximum listening time for the infant. The experiment was paused if the infant became fussy or started crying. If the infant did not calm down, the experiment was terminated after consultation with the parent. We equated the minimum and maximum exposure for both conditions based on the number of words in the SL condition and meters in the RA condition. Each infant listened to the SL stimuli for a minimum of 2.25 min, corresponding to 150 words or 450 syllables. In the RA condition, the minimum exposure time was 2 min, corresponding to 150 m or 600 beats.

#### 2.3.2 Parents—Tasks at Home

The parents both (if available) individually completed three online tasks at home. They were instructed to complete the tasks individually in a quiet environment, using headphones or speakers.

1. The *Goldsmiths Musical Sophistication Index* (Gold‐MSI: Müllensiefen et al. 2014) translated to Dutch (Bouwer et al. 2016). This questionnaire comprises 38 questions; answers on 7‐point Likert scales range from *disagree* to *agree* and generates a general musicality score per participant. Additional questions concerned the frequency of musical activities conducted by the parent themselves, as well as together with the child (see Appendix A for details). This task was always completed first, as the questionnaire included the digital informed consent form.
2. *Computerized Adaptive Beat Alignment Task* (CA‐BAT: Harrison and Müllensiefen 2018a, 2018b). This task consisted of 25 trials in which the participant listened to music combined with beep tracks in two variants. In one variant, the beep track is synchronous with the rhythm of the music, in the other variant beeps and music are unsynchronized. The participant indicated per trial which of the two tracks had the beeps synchronized with the musical rhythm.
3. Rhythm and Accent sub‐tests of the short *Profile of Music Perception Skills* (PROMS: Zentner and Strauss 2017). In this task, the participant heard eight trials for Rhythm and 10 trials for Accent, in which the same drum rhythm (including louder accented and softer unaccented beats in the Accent sub‐test) is played twice, followed by a third. The participant then indicated whether the third was the same as either the first or second on a 5‐point scale (definitely same, probably same, I do not know, probably different, definitely different).

### 2.4 Analyses

The EEG data were processed through scripting in MATLAB version R2019a (The MathWorks Inc. 2019), using EEGLAB version 2023.1 (Delorme and Makeig 2004), and ERPLAB version 10.04 (Lopez‐Calderon and Luck 2014). Statistical analyses were performed using the software JASP version 0.19.1 (JASP Team 2024).

#### 2.4.1 Infant Data

The infants’ EEG data were re‐referenced offline to the algebraic average of the left and right mastoids upon data importing.^8^ Subsequently, it was bandpass filtered from 0.5 to 20 Hz using a filter order of 2 (roll‐off of 12 dB/oct, 40 dB/dec), and 50 Hz notch filtered. Bad channels identified upon visual inspection of the data (showing frequent noise or drifts) or during data collection were interpolated. Following earlier publications (Choi et al. 2020; Cirelli et al. 2016; Fujioka et al. 2011), we used the *Artifact Blocking* (AB) algorithm in MATLAB (Mourad et al. 2007). Before applying the algorithm, we removed the outer ring of channels^9^ as recommended by Fujioka et al. (2011), leaving 21 channels for further analyses. Artifact correction was performed on continuous data, with the total approach of the AB algorithm and the threshold θ set at ±50 µV (Choi et al. 2020; Fujioka et al. 2011). We manually deleted data that contained large artifacts affecting all channels (e.g., the infant being fussy or pulling on the cap) before applying the algorithm. See the Supporting Information for a log of the data cleaning process.

We time‐locked data segments (epochs) to syllable onsets in the SL condition or beat onsets in the RA condition. Epochs in the SL condition were 9 s long (10 word repetitions; as in Choi et al. 2020). Epochs in the RA condition were 8 s long (10 m repetitions). Epochs were non‐overlapping (Batterink and Choi 2021; Benjamin et al. 2021). For both conditions this entailed that if the infant listened for the minimal exposure time (2.25 min in the SL condition or 2 min in the RA condition), there were 15 epochs extracted. If the infant listened for the full duration of each condition (9 min in SL, or 8 min in RA), 60 epochs were extracted.

We quantified neural entrainment with the *Inter‐Trial Coherence* (ITC; Batterink and Choi 2021; Benjamin et al. 2021), which ranges from 0 to 1. An ITC of 1 indicates perfect neural entrainment to a given frequency, and 0 indicates no neural entrainment to that frequency.

$$ITC\left(f\right)=\frac{1}{N}\left|\sum_{i=1}^{N}e^{i\Phi \left(f,\;i\right)}\right|$$

*N* = Number of epochs

*Φ*(*f*, *i*) = Phase at frequency f and trial i

The ITC was calculated after a Fast Fourier Transform between 0.6 and 10 Hz, yielding a bin width of 0.111 in the SL condition and 0.125 Hz in the RA condition. We extracted the ITC values for four frequencies of interest: the word frequency of 1.1 Hz (ITC_word_) and syllable frequency of 3.3 Hz (ITC_syll_) in the SL condition, as well as the meter frequency of 1.25 Hz (ITC_meter_) and beat frequency of 5 Hz (ITC_beat_) in the RA condition. We centered^10^ the ITCs before statistical analyses.

#### 2.4.2 Parental RA

We extracted the mean score of both parents from the CA‐BAT, the PROMS, and the Gold‐MSI. If only one parent completed the task, we included the score of that parent alone. We then standardized^11^ these scores before statistical analyses. Standardizing predictors in a regression helps interpret coefficients on a common scale, making it easier to compare their relative importance.

#### 2.4.3 Statistical Analyses

We quantified evidence in favor of our hypotheses by calculating the Bayes Factor (*BF*). The higher the *BF_10_* is, the more evidence we have for the alternative hypothesis (H1) over the null hypothesis (H0), and the smaller the *BF_10_*, the more evidence for H0 over H1. We adhered to an inference criterium of *BF_10_* > 3, which indicates moderate evidence in favor of H1 (Jeffreys 1961). Correspondingly, a *BF_10_* < 1/3 indicates moderate evidence in favor of H0.

##### 2.4.3.1 Correlation ITC_word_ and ITC_meter_ (RQ1)

**FIGURE 5:**
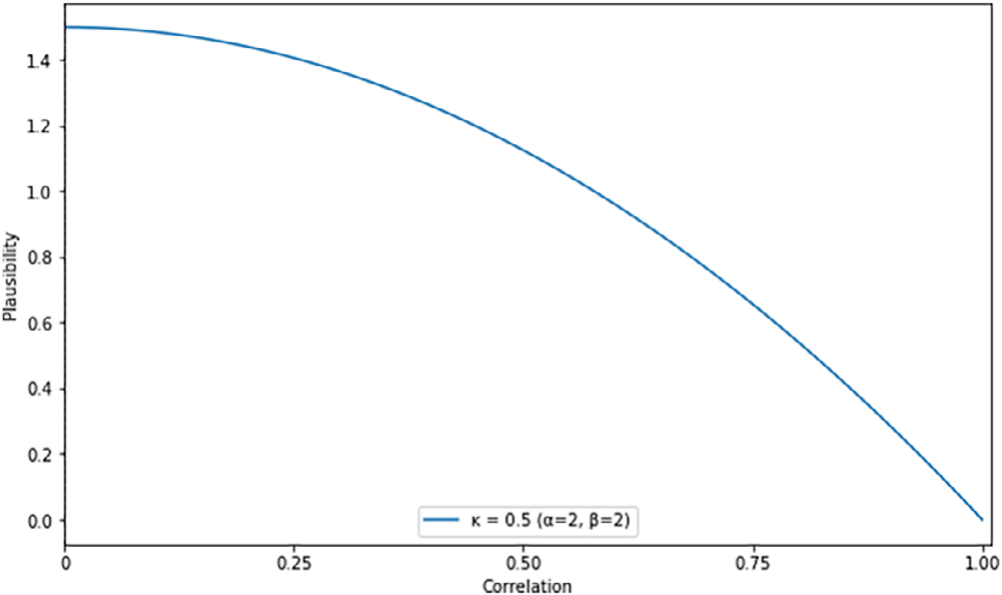
Prior distribution for the one‐sided correlation in JASP.

To answer our first research question, we conducted a one‐sided Bayesian correlation on the ITC_word_ and ITC_meter_. We focused on ITC_word_ and ITC_meter_ because words and meters are hierarchically at corresponding levels (syllables form word in SL and beats form meters in RA; Figure 2). Importantly, ITC_word_ and ITC_meter_ encompass entrainment to different frequencies due to differing frequencies of the stimuli (Section 2.2). Therefore, entrainment effects cannot be attributed to specific input frequencies. The prior for correlations in JASP is described by a beta‐distribution centered around zero and with a width parameter (ĸ). In the case of a one‐sided test, the distribution is truncated at zero, retaining only the positive values. The width is inversely related to the α and β parameters of the beta distribution, which are equal in this case. We adhered to ĸ = 0.5, yielding a distribution beta(2,2). This is a weakly informative prior that places less weight on large effect sizes (Figure 5).

Sensitivity analyses assess the correlation against a range of possible priors. In JASP, this is implemented. If the result does not qualitatively change when the prior changes, the result is robust. We also looked at the incremental analysis of the data, where JASP cumulatively plots the evidence for H1/H0 as the sample size increases. An increasing pattern then supports the evidence for H1.

##### 2.4.3.2 Predicting Infant RA and SL With Parental RA (RQ2 and RQ3)

To assess if parental RA predicted infant RA (RQ2) and SL (RQ3), we performed Bayesian regression analyses, using Bayesian Model Averaging (BMA; Heo and van de Schoot 2020; van den Bergh et al. 2021). BMA provides parameter estimates after considering all the possible models with all possible combinations of the predictors we specified. It selects the best model with the predictors that best fit the data, as parameter estimations based on a single model could give misleading results. We followed the methodology for BMA as described by van den Bergh et al. (2021). The dependent variables of our regression models were the ITC_meter_ (RQ2) and ITC_word_ (RQ3). Predictors were the parents’ scores on the CA‐BAT, PROMS, and Gold‐MSI. Infant age was added as a covariate.^12^

We first tested for (multi)collinearity between our predictors. As van den Bergh et al. (2021) note, collinear predictors could independently improve the model but worsen it together. In our case, the CA‐BAT and PROMS aim to measure the same construct of “rhythmic ability,” and the Gold‐MSI measures the related construct of “musicality.” We looked at the correlations between these variables and calculated the Variance Inflation Factor (VIF). For the correlations, we again adhered to the prior κ = 0.5. Indications for collinearity are Pearson's correlation coefficients above 0.80, and VIFs larger than 10 (Field et al. 2012).

For BMA, one needs to specify two priors. The first prior is the *model prior*, representing the prior belief that some models are more likely than others. However, as we had no prior evidence that our tests of parent RA were linked to infant RA, we considered all models to be equally likely. Therefore, we adhered to the neutral choice of the uniform distribution as the model prior (Heo and van de Schoot 2020; van den Bergh et al. 2021).

The second prior represents the distribution for the regression coefficients: the *regression prior*. We adhered to the default *Jeffreys‐Zellner‐Siouw* (JZS) prior. This is a multivariate Cauchy distribution on the beta coefficients of the model. The scale determining the width of the JZS prior is defined by *r*. We adhered to the default choice in JASP: $r=\sqrt{\frac{1}{8}\;}=0.354$. This is a “medium” scale for the JZS prior (van den Bergh et al. 2021). We determined which model was the best model, and what the relative contribution of each predictor was given the data, by looking at the posterior inclusion probabilities of the predictors (van den Bergh et al. 2021).

Sensitivity analyses encompassed varying both the model and regression priors to determine the robustness of the outcomes. Regarding the model prior, robustness was determined with a beta‐binominal model prior, adhering to the default settings α = β = 1 (following van den Bergh et al. 2021). This prior assigns equal probability to models with the same number of predictors. With regard to the regression prior, we repeated the analyses with “wide” ($r=\sqrt{\frac{1}{4}\;}=0.5)$, and “ultrawide” ($r=\sqrt{\frac{1}{2}\;}=0.707$) scales for the JZS prior (following van den Bergh et al. 2021).

#### 2.4.4 Exploratory Analyses

##### 2.4.4.1 Correlating Infant Neural Entrainment to Syllables and Beats, as Well as Age

We exploratively correlated both the ITC_word_ and ITC_meter_ with infant age, under the same methodology and prior as RQ1 (Section 2.4.3.1.). We also exploratively correlated ITC_syllable_ and ITC_beat_ with each other, as well as with infant age under the same prior.

##### 2.4.4.2 Questionnaire Data—Analysis of Musical Engagement on RA and SL

In the questionnaire, four questions were added to estimate parent musical engagement (pME) and parent–child joint musical engagement (pcME). Parents were asked how often they listen to music, sing, dance, or perform other musical activities by themselves, and how often they did this together with their child. These questions appeared on four‐point scales (never, rarely, sometimes, often; see Appendix A). We exploratively included the sum of both parents’ scores on these questions as predictors in BMA analyses following the exact same methodology as 2.4.3.2. We took the sum instead of the mean because the questions were on an ordinal scale and taking the sum makes them more linear (i.e., summed scores could range from 4 to 16 per variable). If only one of the parents completed the questionnaire (*N *= 3), the score of that parent was excluded from this analysis.

## 3 Results

### 3.1 Infant EEG Results (RQ1)

**FIGURE 6:**
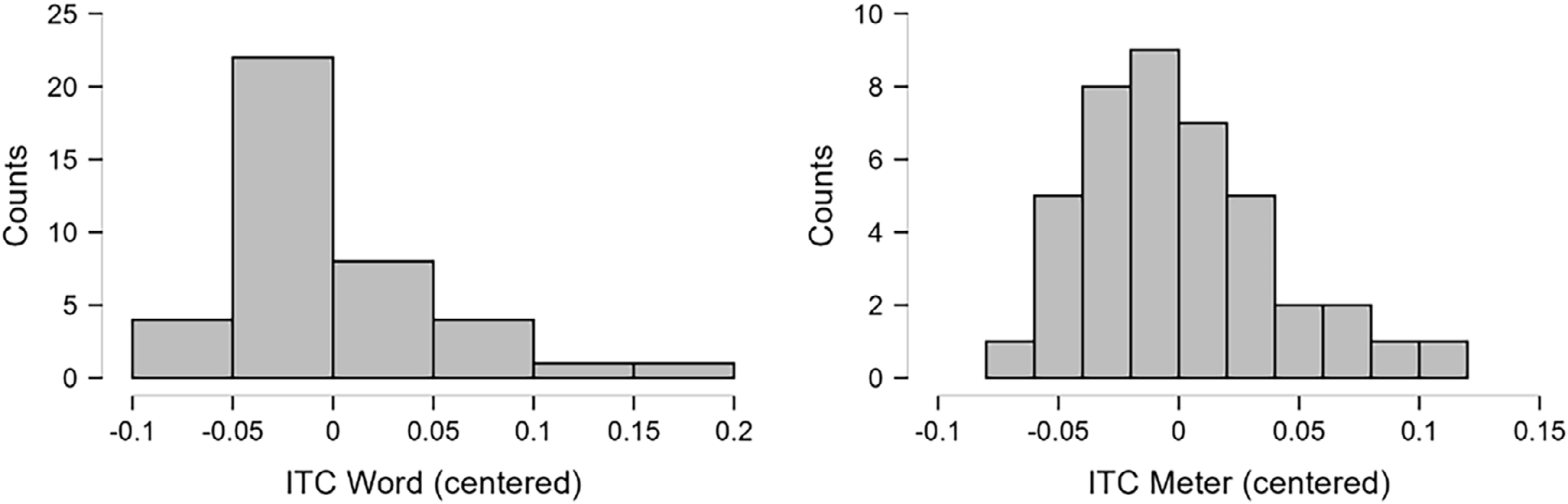
Distributions of ITC_word_ and ITC_meter_. Both variables are centered.

**FIGURE 7:**
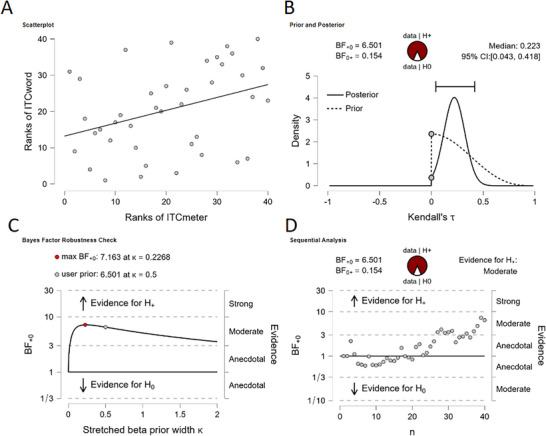
Results for the Bayesian Correlation analysis in JASP between ITC_word_ and ITC_meter_. A) Scatterplot shows the correlation plot; B) Prior and Posterior plots the correlation against the prior; C) Bayes Factor Robustness Check plots the *BF_10_* against the possible range of priors; and D) the Sequential Analysis plots the *BF_10_* against the cumulative data. Figure from JASP.

We evaluated ITC_word_ and ITC_meter_ for normality with a Shapiro–Wilk test (alpha level: *p *< 0.05), which indicated that the ITC_word_ was not normally distributed (Figure 6; ITC_word_: *W* = 0.90, *p* = 0.002; ITC_meter_: *W* = 0.95, *p* = 0.08). Therefore, we decided to calculate Kendall's tau‐b (*τb*) correlation coefficient. We found evidence for a positive correlation between ITC_word_ and ITC_meter_ (*τb* = 0.25, *BF_10_* = 6.50, 95% CI [0.04, 0.42]; Figure 7). This indicates that infants who showed more neural entrainment to the words in the SL condition also showed relatively more neural entrainment to the meter in the RA condition. Sensitivity analyses indicated that the result is robust against the prior, as the *BF_10_* does not drop below 3 when the prior κ is increased (Figure 7C). Furthermore, evidence in favor of H1 is increasing as the amount of data increases (Figure 7D).

To establish whether this correlation was specific to the frequencies of interest, and did not appear at unrelated frequencies, we additionally performed correlations on 11 frequency pairs between the SL and RA conditions, from the word/meter frequency to the first harmonics (Table 1). Due to the difference in stimulus frequencies in the RA and SL conditions, as well as different epoch lengths (Section 2.4.1.), the frequency bins differed in size; 0.111 Hz steps in the SL condition, and 0.125 Hz steps in the RA condition. In both conditions, there were exactly 11 steps from the word/meter frequency up to the first harmonic. Therefore, we performed the same one‐sided correlations on these 11 frequency pairs as for the word/meter frequencies, under the same prior. None of these other correlations reached *BF_10_* > 3. Furthermore, they were not robust against prior variations. See Table 1 for a summary of these results, as well as the Supporting Information for the sensitivity analyses. In sum, the correlation in our data is specific to neural entrainment to words and meters.

**TABLE 1:** Kendall's tau‐b (τb) correlations between frequency pairs in RA and SL conditions, from word/meter (RQ1) up to the first harmonic.

			*95% CI*		
Frequency pair	*τb*	*BF_10_*	*Lower*	*Upper*	Robust against *κ*?
RA1.250–SL1.111 (RQ1)	0.25	6.50^*^	0.04	0.42	Yes
RA1.375–SL1.222	0.06	0.50	0.01	0.27	No
RA1.500–SL1.333	−0.15	0.14	0.00	0.16	No
RA1.625–SL1.444	0.10	0.73	0.01	0.30	No
RA1.750–SL1.556	0.19	2.33	0.02	0.37	No
RA1.875–SL1.667	0.02	0.34	0.00	0.24	No
RA2.000–SL1.778	−0.06	0.20	0.00	0.20	No
RA2.125–SL1.889	0.12	0.91	0.01	0.31	No
RA2.250–SL2.000	0.03	0.38	0.00	0.25	No
RA2.375–SL2.111	0.04	0.40	0.00	0.25	No
RA2.500–SL2.222 (1st harmonics)	0.19	2.33	0.02	0.37	No

*Note*: The alternative hypothesis specifies that the correlation is positive. The prior used for all correlations was *κ *= 0.5.

* *BF_10_* > 3.

### 3.2 Results of Predicting Infant RA and SL With Parental RA (RQ2 and RQ3)

#### 3.2.1 Tests for Collinearity

We first tested the CA‐BAT, PROMS, and Gold‐MSI for normality with a Shapiro–Wilk test, which indicated that the Gold‐MSI was not normally distributed (Gold‐MSI: *W* = 0.90, *p* = 0.001; CA‐BAT: *W* = 0.95, *p* = 0.06; PROMS: *W* = 0.98, *p* = 0.62). Therefore, we calculated Kendall's tau‐b (*τb*) correlation coefficients. When using Pearson's, an *r *> 0.80 indicates collinearity (Field et al. 2012); this converts to *τb *> 0.59 (Gilpin 1993).

We found a positive correlation between the PROMS and CA‐BAT (*τb* = 0.20, *BF_10_* = 3.12, 95% CI [0.03, 0.37]). However, this was not robust to the prior. We also found a positive correlation between the Gold‐MSI and CA‐BAT (*τb* = 0.32, *BF_10_* = 37.32, 95% CI [0.09, 0.47]), which was robust. Still, both correlations did not indicate collinearity (*τb's *< 0.59). There was inconclusive evidence for a correlation between the PROMS and Gold‐MSI (*τb* = 0.07, *BF_10_* = 0.51, 95% CI [0.01, 0.27]). We also looked at the VIF to measure collinearity. The VIF for all variables was around 1, so this test did not indicate collinearity either. Finally, there was robust evidence against (*BF_10_* < 0.33) correlations between infant age, the PROMS, and the Gold‐MSI. The CA‐BAT and infant age were close to evidence for no correlation (*BF_10_* = 0.397). Taken together, this indicates that the tasks for parental RA were not collinear, and older infants did not have parents with more musical or RA in our sample.

#### 3.2.2 RQ2: Does Parental RA Predict Infant RA?

The residuals deviated from a normal distribution, violating an assumption of regression (Field et al. 2012). A log‐transformation of ITC_meter_ improved the normality of the residuals (see the Q‐Q plots in JASP in the Supporting Information). Results of the BMA analysis showed that none of the predictors had a large enough effect on infant RA to be included in the best model. The best model was the null model consisting of only the intercept. Table 2 shows the model‐averaged posterior summary of the predictors.

**TABLE 2:** Model‐averaged posterior summary for the linear regression coefficients of parental RA on infant RA (RQ2).

						95% Credible interval	
Coefficient	*P*(incl)	*P*(incl|data)	*BF_inclusion_*	Mean	SD	Lower	Upper
Intercept	1.00	1.00	1.00	0.01	0.02	−0.04	0.04
CA‐BAT	0.50	0.31	0.45	−0.00	0.01	−0.03	0.02
PROMS	0.50	0.33	0.48	−0.00	0.01	−0.03	0.02
Gold‐MSI	0.50	0.29	0.41	0.00	0.01	−0.02	0.03
Infant age	0.50	0.29	0.40	0.00	0.01	−0.02	0.03

*Note*: The leftmost column denotes the predictor. *P*(incl) shows the prior inclusion probabilities (these are identical due to the uniform prior). *P*(incl|data) indicates the posterior probabilities of including the predictor in the model. *BF_inclusion_* indicates the BF resulting from the change from prior to posterior inclusion probabilities. Mean and SD represent the posterior means and standard deviations of the predictor after model averaging. The final two columns represent the 95% credible interval for the parameters.

#### 3.2.3 RQ3: Does Parental RA Predict Infant SL?

**FIGURE 8:**
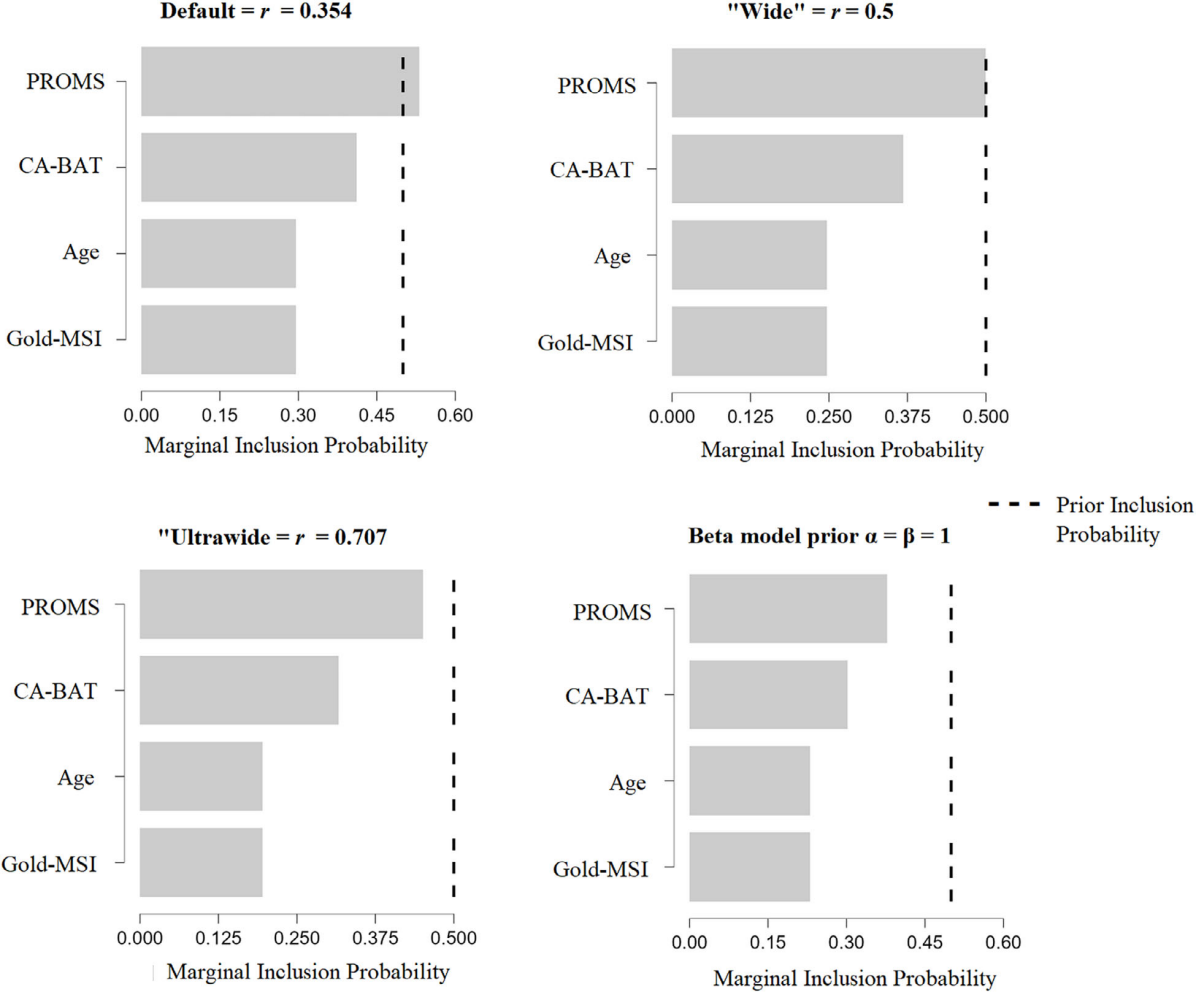
Results of the sensitivity analysis for the posterior inclusion probabilities of the predictors.

The residuals for this analysis also deviated from a normal distribution. Log transformation of ITC_word_ improved this. Results of the BMA analysis (Table 3) showed that the best model included a negative effect of the PROMS, but the evidence for this effect was inconclusive (*BF_inclusion_* = 1.13). The effect was also not robust against variations in both the model and regression priors (Figure 8).

**TABLE 3:** Model‐averaged posterior summary for the linear regression coefficients of parental RA on infant SL *(RQ3)*.

						95% Credible interval	
Coefficient	*P*(incl)	*P*(incl|data)	*BF_inclusion_*	Mean	SD	Lower	Upper
Intercept	1.00	1.00	1.00	−0.00	0.03	−0.06	0.04
PROMS	0.50	0.53	1.13	−0.02	0.02	−0.06	0.01
CA‐BAT	0.50	0.41	0.70	−0.01	0.02	−0.06	0.01
Gold‐MSI	0.50	0.30	0.42	0.00	0.01	−0.03	0.03
Infant age	0.50	0.30	0.42	0.00	0.01	−0.03	0.04

*Note*: The leftmost column denotes the predictor. *P*(incl) shows the prior inclusion probabilities (these are identical due to the uniform prior). *P*(incl|data) indicates the posterior probabilities of including the predictor in the model. *BF_inclusion_* indicates the BF resulting from the change from prior to posterior inclusion probabilities. Mean and SD represent the posterior means and standard deviations of the predictor after model averaging. The final two columns represent the 95% credible interval for the parameters.

#### 3.2.4 Results of Exploratory Analyses

##### 3.2.4.1 Exploratory Correlations of Infant Neural Entrainment

There was evidence for no correlations between all indices of neural entrainment and infant age (*BF_10_* < 0.33; Table 4). Furthermore, we did not find evidence for a correlation between ITC_syllable_ and ITC_beat_ (*τb* = 0.09, *BF_10_* = 0.62, 95% CI [0.01, 0.29]).

**TABLE 4:** Results of the exploratory correlation analyses between infant neural entrainment and age.

Infant age	ITC_word_	ITC_meter_	ITC_syllable_	ITC_beat_
*τb*	−0.03^*^	−0.01^*^	−0.01^*^	−0.02^*^
*BF_10_*	0.24	0.28	0.27	0.27
95% CI	[0.00, 0.21]	[0.00, 0.22]	[0.00, 0.22]	[0.00, 0.22]

*Note*: For all tests, the alternative hypothesis specifies that the correlation is positive. Prior: *κ *= 0.5.

* *BF_10_* < 1/3.

##### 3.2.4.2 Effects of Musical Engagement on Infant RA

We checked for collinearity between parental musical engagement (pME; Section 2.4.4.2.), and parent–child musical engagement (pcME). Results indicated a positive correlation between these variables (*τb* = 0.27, *BF_10_* = 4.73, 95% CI [0.04, 0.43]), but not collinearity. The VIF was 1.05, and also did not indicate collinearity. Table 5 presents the results of the BMA analysis predicting infant RA (using the log‐transformed ITC_meter_) with these variables. Both predictors yielded inconclusive BFs. However, sensitivity analyses revealed that both predictors were always included in the best model, except for pcME under the “ultrawide” JZS prior. The effect of pME was in the negative direction. On the other hand, the effect of pcME was positive.

**TABLE 5:** Model‐averaged posterior summary for the linear regression coefficients of parental and parent–child musical engagement on infant RA.

						95% credible interval	
Coefficient	*P*(incl)	*P*(incl|data)	*BF_inclusion_*	Mean	SD	Lower	Upper
Intercept	1.00	1.00	1.00	0.02	0.02	−0.03	0.05
Parent musical engagement	0.50	0.65	1.86	−0.02	0.02	−0.07	0.00
Parent–child musical engagement	0.50	0.55	1.21	0.02	0.02	−0.00	0.07

*Note*: The leftmost column denotes the predictor. *P*(incl) shows the prior inclusion probabilities (these are identical due to the uniform prior). *P*(incl|data) indicates the posterior probabilities of including the predictor in the model. *BF_inclusion_* indicates the BF resulting from the change from prior to posterior inclusion probabilities. Mean and SD represent the posterior means and standard deviations of the predictor after model averaging. The final two columns represent the 95% credible interval for the parameters.

##### 3.2.4.3 Effects of Musical Engagement on Infant SL

We conducted the same analysis as in 3.2.4.2. with the log‐transformed ITC_word_ as the dependent variable (Table 6). Results revealed that the null model was the best model. This model also showed a violation of the assumption that the residuals are normally distributed, even though the dependent variable was already log‐transformed.

**TABLE 6:** Model‐averaged posterior summary for the linear regression coefficients of parental and parent–child musical engagement on infant SL.

						95% credible interval	
Coefficient	*P*(incl)	*P*(incl|data)	*BF_inclusion_*	Mean	SD	Lower	Upper
Intercept	1.00	1.00	1.00	0.00	0.03	−0.05	0.05
Parent musical engagement	0.50	0.27	0.37	−0.00	0.01	−0.04	0.03
Parent–Child musical engagement	0.50	0.45	0.82	0.02	0.03	−0.01	0.07

*Note*: The leftmost column denotes the predictor. *P*(incl) shows the prior inclusion probabilities (these are identical due to the uniform prior). *P*(incl|data) indicates the posterior probabilities of including the predictor in the model. *BF_inclusion_* indicates the BF resulting from the change from prior to posterior inclusion probabilities. Mean and SD represent the posterior means and standard deviations of the predictor after model averaging. The final two columns represent the 95% credible interval for the parameters.

## 4 Discussion

We evaluated the hypothesis that there is a relationship between SL performance and RA in infants between 6 and 9 months of age. We measured both SL and RA using neural entrainment, which is a direct and online parameter that is sensitive to individual variability and suitable for the infant population. We also (behaviorally) investigated parental RA as a possible predictor for infant RA and SL, and exploratively investigated parent solo and parent–child joint musical engagement (pME and pcME) as predictors of infant RA and SL.

### 4.1 Statistical Evidence for a Correlation Between Neural Measures of SL and RA

We hypothesized that SL and RA rely on overlapping neural mechanisms (Section 1.3.). Our hypothesis was built on several research findings that, first, established correlations between RA and language development more generally (see Fiveash et al. 2021; and Ladányi et al. 2020 for review), and secondly provided evidence for an overlap in neurocognitive mechanisms underlying both RA and SL (Assaneo et al. 2019). We found statistical evidence that infants’ RA—indicated by the amount of neural entrainment to the meter of a musical rhythm—was correlated with their amount of neural entrainment to words in the SL speech segmentation task. This result supports our hypothesis for a relationship between SL and RA in infancy. Importantly, we did not find evidence for SL‐RA correlations in an additional sensitivity analysis of paired frequencies that were not word/meter‐related and thus not frequencies of interest. This indicates that we found a relationship specifically between neural processing of meters and words, which are on corresponding hierarchical levels (Figure 2). Furthermore, we found Bayesian evidence for the absence of associations between neural entrainment and infant age in our data, pointing to neural entrainment as a capacity that is not modulated by age in the 3‐month age group we investigated.

Surprisingly, the exploratory analyses did not yield evidence for correlated beat and syllable entrainment. Previous studies on the relationship between language and music did find such relations between RA and linguistic auditory processing (review: Fiveash et al. 2021). However, the beat in our RA condition was presented less consistently than the syllables in the SL condition. This is due to the syncopated nature of the rhythm, causing the beat frequency to encompass both the beats and silent periods. In contrast, the syllables in the SL condition were consistently presented, yielding a steadier auditory stimulation than the beat stimuli in the RA condition. This is a trade‐off of measuring meter processing as an index of RA. In the case of the stimuli employed in the current study, the syncopation enables the meter interpretation. If the rhythmic stimulus would be presented by a continuous beat without silent periods, any meter interpretation is possible (Nozaradan et al. 2011). We conjectured meter processing to be analogous to word‐level processing in the SL condition, as meters and words are on corresponding levels in the beat‐meter or syllable‐word hierarchies (Figure 2). Another advantage of the rhythmic stimulus we employed was that it was monotonous, which is similar to the SL stimuli. It may be that a meter interpretation can also be formed by using different tones instead of silent periods. However, low‐tone rhythmic stimuli invoke more (neural) processing of the meter compared to high‐tone stimuli, congruent with bass instruments generally delivering rhythmic information and inducing sensory‐motor entrainment in musical traditions around the globe (Lenc et al. 2018; Lenc et al. 2022). Future research could investigate whether neural processing of syllables and beats is related if both are presented in more similar ways.

In addition, we recommend further research to investigate whether these results generalize to speech streams with variable syllable durations, as in natural spoken language (Peelle and Davis 2012; Poeppel and Assaneo 2020). A recent study found behavioral evidence in adults for intact SL in arhythmic conditions (Gómez Varela et al. 2024). To our knowledge, this has not yet been investigated with neural entrainment. Investigating neural entrainment to stimuli with variable durations is challenging, as it is difficult to establish one frequency of interest. For instance, Gómez Varela et al. (2024) determined the syllable durations in their semi‐rhythmic and arhythmic conditions with probability density functions. In the case of the arhythmic condition, this was a uniform distribution between 125 and 500 ms. This does not create one syllable frequency, but instead a syllable frequency that fluctuates randomly between 2 and 8 Hz. In the case of word segmentation SL research using neural entrainment, future studies could overcome this “frequency challenge” by focusing on neural entrainment to the word frequency. If the total duration of the words is constant, there will still be a corresponding word frequency. In other words, if the word frequency is 1.1 Hz as in the current study, the sum of the durations of the three syllables making up the words should always be 900 ms, but the durations of these individual syllables can be varied. Further research could delve into this topic, and is recommended to additionally investigate whether SL in arhythmic conditions also relates to RA.

### 4.2 No Effects of Parental RA, But Active Engagement With Music Yields Promising Results

We found no evidence of parental RA predicting infant RA as indexed by meter processing. Likewise, we found no effects of parental RA on the SL ability of the infants. In both cases, none of the tasks measuring RA or musicality of the parents could be included in the best models after sensitivity analyses. However, the exploratory analyses indexing pME and pcME did point to interesting avenues for future research when infant RA was the dependent variable (Section 3.2.4.2). Although both these composite measures taken from the questionnaire did not yield sufficient evidence for H1 (i.e., the Bayes Factors were inconclusive), they were suggestive of a negative effect of pME, in contrast to a positive effect of pcME. These inconclusive results are probably due to the limited statistical sample size of the current study. An explanation for a negative effect of pME could be that these musical activities tend to be conducted in the absence of the infant (e.g., in a music school or choir). Our results do not support the hypothesis that musical skills of parents contribute directly to their infants’ RA. On the other hand, pcME does seem to have a positive effect on infant RA, which is in line with earlier research. For instance, Cirelli et al. (2016) found that 7‐month‐old infants enrolled in music classes showed enhanced neural entrainment to meter, compared to infants who did not attend these classes. Engagement with music at this early age could thus already positively influence infants’ RA.

Moreover, Boyne et al. (2025) showed that parental singing and the presence of music in the home were positive predictors of infant vocabulary and gestures. It appeared that the social motivation (indexing the degree of interest and motivation in social interactions) of parents, was a significant predictor of the home music environment, but not parental musical training. Boyne et al. (2025) also found a positive relationship between infant RA and vocabulary. Although we did not find evidence for a direct relationship between pcME and SL in our exploratory analyses, we did find promising results regarding effects of the musical environment on infant RA, as outlined above. As our results support the hypothesis of related neurocognitive underpinnings of infant RA and SL, we hypothesize that infants’ active engagement with music positively influences their RA, which in turn is a positive predictor of their SL ability and early word segmentation. Future studies should investigate this hypothesis further, preferably in longitudinal designs. In sum, there is a link between RA and language development that is already present in infancy, and infant RA is likely to be aided by social interactions around music.

### 4.3 Neurological Development of the Dorsal Stream in Infancy

Our hypothesis connecting individual differences in SL to RA was based on studies specifying the neurocognitive functions of the auditory dorsal stream (Section 1.3.2.). Taken together with the heritability of musicality (Gingras et al. 2015; Niarchou et al. 2022), we hypothesized that the dorsal stream is part of the neurological substrate of innate musical ability. However, we found no evidence of parental RA predicting either infant RA or SL. This is in line with Cirelli et al. (2016), who found no effects of parental musical training in infants of 7 months but did find such an effect in older infants of 15 months.

A possible explanation for these results may be found in the neurological development of the dorsal stream. Structural imaging research in newborns showed that the dorsal auditory pathway is not yet fully developed at birth (Brauer et al. 2013; Dubois et al. 2016). The connections from the temporal cortex to the premotor cortex are visible, but the connections to the inferior frontal gyrus are not yet present in newborns. Dubois et al. 2016) found that the dorsal stream was less mature than the ventral stream in early infancy, and that this difference disappeared during the first post‐natal months. The authors submit that this is due to the broadening of infants' input from primarily auditory to additional new visual and motor inputs in the first months of post‐natal life. Since the dorsal stream is involved in sensorimotor integration, it needs to develop quickly during this increase in multi‐modal processing in auditory, visual, and motor regions of the infant brain (see Choi et al. (2023) for review). We would suggest that the aforementioned social interactions around music are among the candidate positive contributors to the development of the auditory dorsal stream.

## 5 Conclusion

To conclude, we found evidence for a relationship between measures of neural entrainment assessing SL and RA in 6‐ to 9‐month‐old infants that was not modulated by infant age. This relationship was specific to neural entrainment at word and meter frequencies, suggesting similarity between processing items at hierarchically corresponding levels. We also tested whether behavioral parental RA was a predictor of child RA or SL and found no evidence that it was. However, indices of parent–child joint musical engagement provide results that pave the way for further studies on this topic.

## Author Contributions

**I. M. (Iris) van der Wulp**: Conceptualization, Methodology, Formal analysis, Visualization, Investigation, Writing – Original Draft. **M. E. (Marijn) Struiksma**: Conceptualization, Methodology, Writing – Review & Editing. **F. N. K. (Frank) Wijnen**: Conceptualization, Writing – Review & Editing, Project administration, Funding acquisition.

## Funding

This work is funded by the Netherlands Organization for Scientific Research (NWO), project number PGW.21.007.

## Ethics Statement

The experiment was approved by the Linguistics Chamber of the Faculty Ethics Assessment Committee of Humanities at Utrecht University (reference number: LK‐ 23‐104‐02).

## Conflicts of Interest

The authors declare no conflicting interests.

## Preregistration

This study was preregistered: https://osf.io/u8pgn

## Supporting information




**Supporting File 1**: desc70116‐sup‐0001‐Appendix‐A.docx
All Supplementary Materials including analysis code for this project are openly available through www.doi.org/10.17605/OSF.IO/A9BDZ

## Data Availability

The data are openly available through https://doi.org/10.24416/UU01‐ZJHU40

## Endnotes

1 Examples of frequently used metrical structures are triple meters (three quarter notes per measure) in a waltz and quadruple meters (four quarter notes per measure) in most pop music.

2 Fractional anisotropy is a measure resulting from Diffusion Tensor Imaging (DTI), that is sensitive to differences in density, size, and myelination of axons, as well as the organizational coherence of fibers, and thus provides an index of the “structural integrity” of white matter (Basser and Pierpaoli, 1996).

3 For more information about cue points, see this manual.

4 These stimuli are openly available at https://osf.io/9wf5u/ called: ‘L_syncopated_200ms_calib75dB_max_short‐term_ff_freq130Hz_onset10_offset50.wav’

5 Genelec 1029A

6 The infants watched a purchased copy of the following video, muted: https://www.youtube.com/watch?v=4ROrW727q_s

7 21,5″ LCD (TN panel) Iiyama B2283HS

8 For seven infants, we used one mastoid as reference in one or both conditions, as the other electrode was pulled off or noisy. See the Supplementary Materials for an EEG cleaning logbook with details per infant.

9 Outer ring channels that were removed: Fp1, Fp2, F7, F8, T7, T8, P7, P8, O1, Oz, O2.

10 Centering was performed by subtracting the mean from the variable. The resulting mean is zero while maintaining the variable's original scale and variance.

11 Standardization was performed by subtracting the mean from the variable, and subsequently dividing that by the standard deviation of the variable.

12 Infant age was recorded in the format *months;days* at the time of the EEG session. To be able to include the infants’ age in our analyses, we converted age to a decimal number. This was done by dividing the days by 30.4 (the average number of days in a month). For example, if an infant's age was 8;05 (8 months and 5 days), the converted numerical age became $\frac{5}{30.4}$+ 8 = 8.16.
