# Supplementary material for: Words and Meters: Neural Evidence for a Connection Between Individual Differences in Statistical Learning and Rhythmic Ability in Infancy
Source: Dev Sci. 2025 Dec 31;29(2):e70116. doi: 10.1111/desc.70116 (PMC12754635; doi:10.1111/desc.70116)
Supplement: Supplementary file 1 — Supporting File 1: desc70116‐sup‐0001‐Appendix‐A.docx All Supplementary Materials including analysis code for this project are openly available through www.doi.org/10.17605/OSF.IO/A9BDZ [file DESC-29-e70116-s001.docx]

**Appendix A**

The questionnaire, filled in by the parents, contained questions about their:

- Age
- Education in the form of highest attended level; five levels from low to high:
  - Primary school (Dutch: *basisschool*)
  - High school (Dutch: *middelbare school*)
  - Intermediate vocational training (Dutch: *MBO*)
  - Applied university (Dutch: *HBO*)
  - University (Dutch: *Universiteit/WO*)
- Native language(s)
- Other languages they speak at home with their child
- If they play a musical instrument, sing, rap, or dance, with six levels and participants can select multiple answers. Participants are regarded as musicians if they select one or more of the first five answers, and as non-musicians if they select answer option six only:
  - I play one (or more) musical instrument(s)
  - I sing
  - I dance
  - I rap
  - I do something else with music, namely: (textbox)
  - I do not actively take part in making music
- If they are a professional musician or dancer
  - Yes/no.
- How often they listen to music, with four levels:
  - Often: ≥ once a week
  - Sometimes: 1-4 times a month
  - Rarely: < once a month
  - Never
- How often they sing/dance/perform other musical activities, with four levels:
  - Often: ≥ once a week
  - Sometimes: 1-4 times a month
  - Rarely: < once a month
  - Never
- How often they listen to music with their child, with four levels:
  - Often: ≥ once a week
  - Sometimes: 1-4 times a month
  - Rarely: < once a month
  - Never
- How often they sing/dance/perform other musical activities with their child, with four levels:
  - Often: ≥ once a week
  - Sometimes: 1-4 times a month
  - Rarely: < once a month
  - Never
- Musicality, measured with the *Goldsmiths Musical Sophistication Index* (Gold-MSI: Müllensiefen et al., 2014) translated to Dutch (Bouwer et al., 2016). This questionnaire comprises 38 questions; answers on 7-point Likert scales range from *disagree* to *agree*.
  - For example: *I spend a lot of my free time doing music-related activities.*
    1. Completely disagree
    2. Strongly disagree
    3. Disagree
    4. Neither agree nor disagree
    5. Agree
    6. Strongly agree
    7. Completely agree
